# Supplementary material for: Inhibition of A2AR alleviates adenosine-mediated suppression of plasma cell differentiation
Source: Front Immunol. 2025 Dec 15;16:1702402. doi: 10.3389/fimmu.2025.1702402 (PMC12745401; doi:10.3389/fimmu.2025.1702402)
Supplement: Supplementary file 1 [file DataSheet1.pdf]

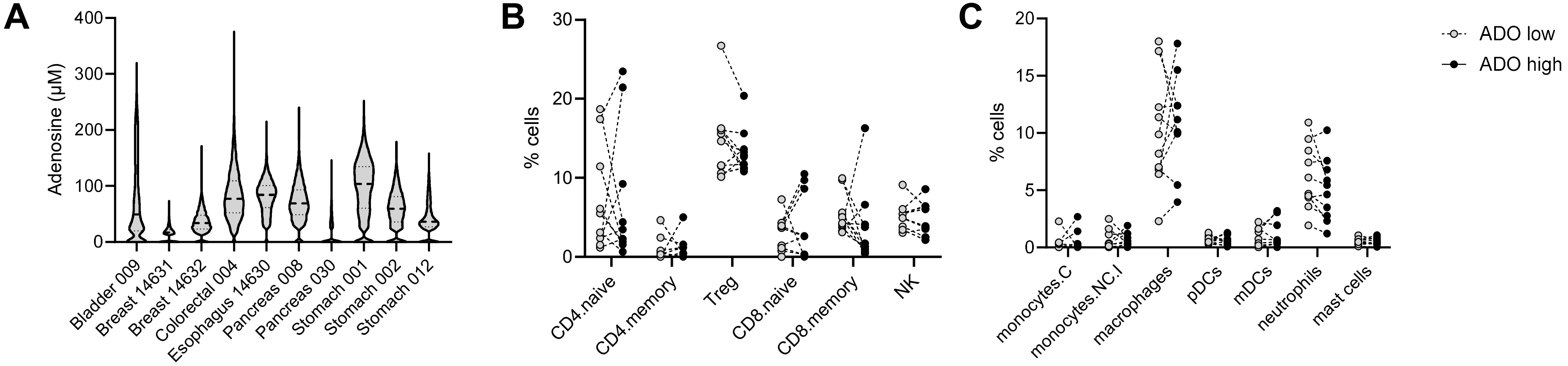

Supplementary Figure 1. A: Violin plot of adenosine distribution in the selected ROIs. **B, C**: Estimated percentages of lymphoid (**B**) and myeloid (**C**) cell populations in adenosine-low and –high ROIs. Each symbol represents one sample, analysis by paired t test. All p>0.05 (not displayed).

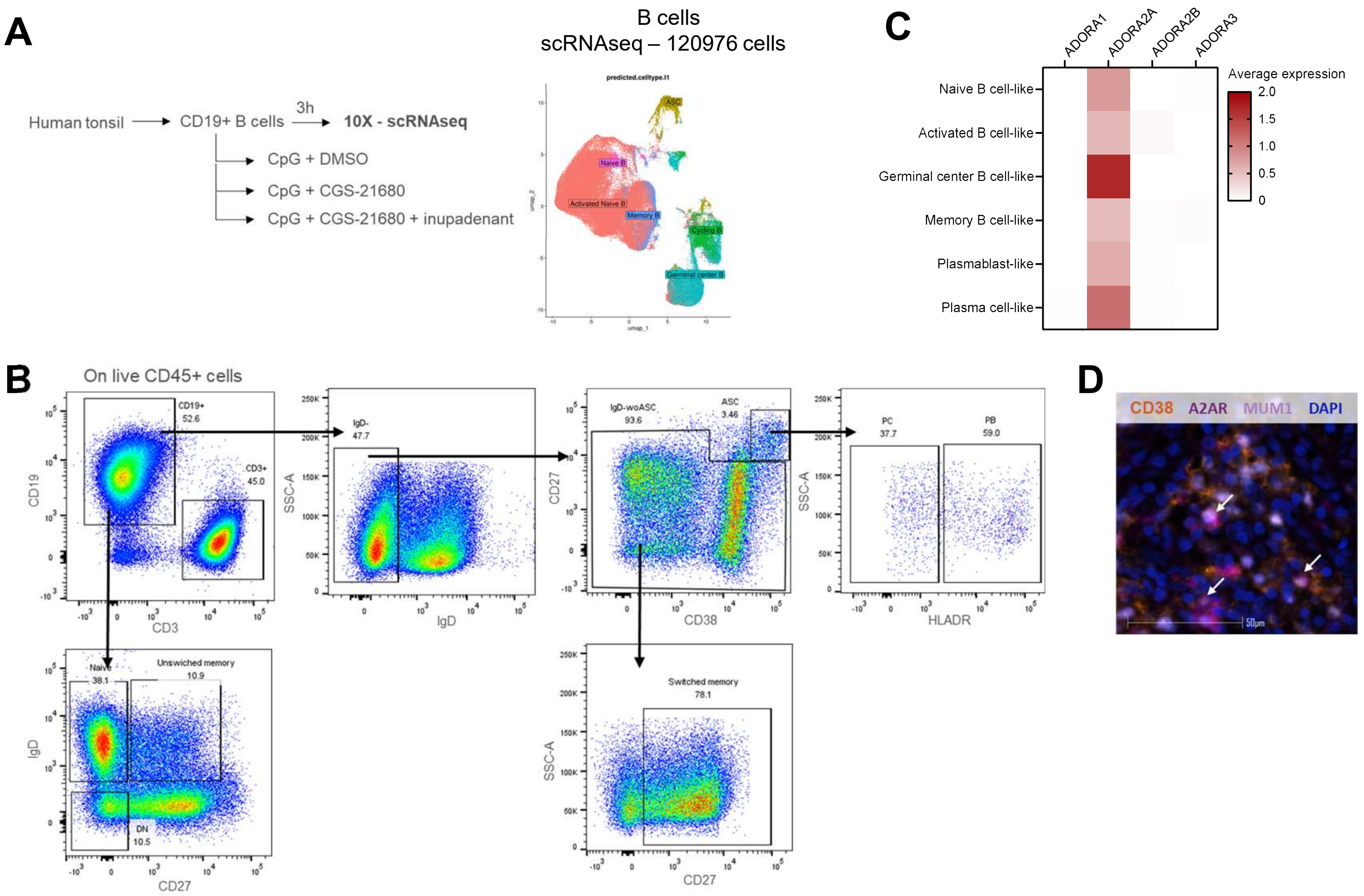

Supplementary Figure 2. A: Schematic representation of scRNAseq experiment (left); UMAP plot of human tonsillar B cells scRNAseq data. **B**: Gating strategy for flow cytometry analysis of different B cell populations. One representative tonsil sample is shown. ASCs (antibody secreting cells): CD19+IgD-CD38++CD27++; PB (plasma blasts): CD19+IgD-CD38++CD27++HLADR+; PC (plasma cells): CD19+IgD-CD38++CD27++HLADR-; naïve B cells: CD19+CD27-IgD+; DN (double negative): CD19+CD27-IgD-; switched memory: CD19+IgD-CD27+w/oCD27++CD38++. **C**: Average expression of ADORA genes across different B cell subpopulations in human melanomas by scRNAseq. **D**: Representative image of merged staining for A2AR, MUM1 and CD38. Arrows indicate cells positive for the three markers.

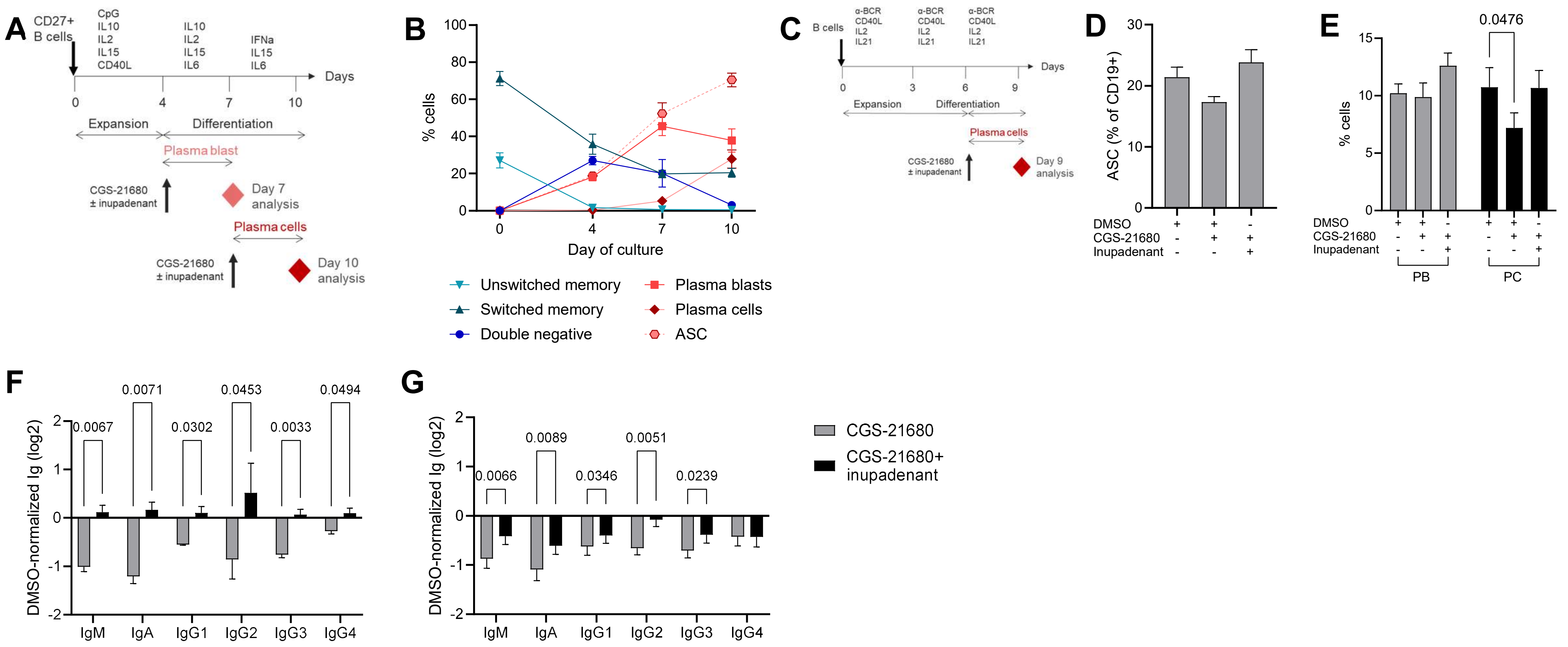

Supplementary Figure 3. A: Schematic representation of *in vitro* differentiation methods of peripheral blood CD27+ purified memory. **B**: Flow cytometry analysis of cultured cells in the absence of CGS-21680 and inupadenant at day 0, 4, 7 and 10. **C-E**: Schematic representation of an alternative method to differentiate peripheral blood CD19+ B cells into PBs and PCs (**C**), and flow cytometry analysis of the effect of CGS-21680 ± inupadenant on the frequency of ASC (**D**) including PB and PC (**E**) at day 10. **F, G**: Effect of CGS-21680 ± inupadenant on immunoglobulin (Ig) secretion at day 7 (**F**) and day 10 (**G**) of method described in A. Ig were quantified with Legendplex, normalized to DMSO condition and log2-transformed. Data presented as mean ± SEM of N=4 healthy donors. P from ANOVA adjusted for multiple comparison. Only significant p values ($p<0.05$) are displayed.

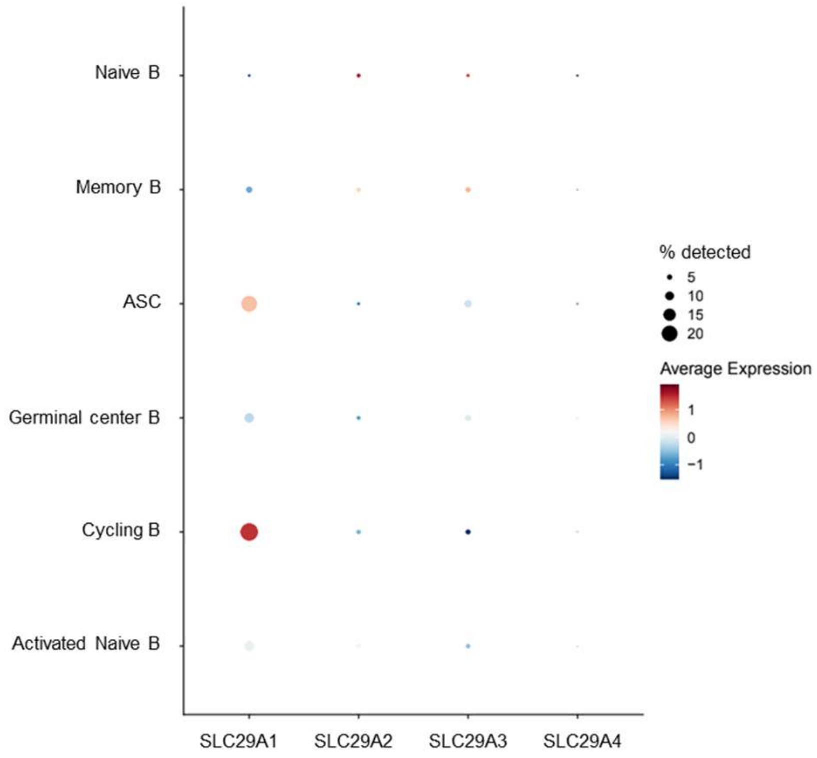

Supplementary Figure 4. Expression of genes encoding for equilibrative nucleoside transporters in B cells.
Average expression of SLC29 genes across different B cell subpopulations in a human tonsil by scRNAseq (DMSO condition is shown).
