# Supplementary material for: Inhibition of A2AR alleviates adenosine-mediated suppression of plasma cell differentiation
Source: Front Immunol. 2025 Dec 15;16:1702402. doi: 10.3389/fimmu.2025.1702402 (PMC12745401; doi:10.3389/fimmu.2025.1702402)
Supplement: Supplementary file 3 [file DataSheet3.pdf]

Antigen	Fluorochrome	Clone	Dilution	Provider
**Sorting of B cell subsets**				
CD45	FITC	REA747	1:200	Miltenyi Biotech
CD3	PerCP	REAL104	1:100	Miltenyi Biotech
CD38	Vio Bright V423	REA572	1:100	Miltenyi Biotech
CD19	APC	REA675	1:200	Miltenyi Biotech
IgD	Vio Bright R720	REA740	1:100	Miltenyi Biotech
CD27	PE	REA499	1:100	Miltenyi Biotech
HLADR	PE-vio 770	REA805	1:100	Miltenyi Biotech
Viability dye	eF780		1:500	Invitrogen
***In vitro* differentiation of B cells**				
CD38	BV421	HIT2	1:100	BD Biosciences™
IgD	BV510	IA6-2	1:100	BD Biosciences™
CD3	PerCP-Cy5.5	UCHT1	1:50	BD Biosciences™
CD27	PE	L128	1:25	BD Biosciences™
HLA-DR	PE-Cy7	L243	1:200	BioLegend
CD19	AF647	HIB19	1:100	BioLegend
CD19	BV711	HIB19	1:100	BioLegend
Viability dye	eF780		1:500	Invitrogen
Ki67	AF488	Ki-67	1:50	BioLegend
CD45	BUV395	HI30	1:500	BD Biosciences™
**CREB phosphorylation assay**				
CREB/AFT-1	PE	pS133/S63	1:6.25	BD Biosciences™
CD4	BV786	SK3	1:100	BD Biosciences™
CD8a	Alexa Fluor 488	HIT8a	1:100	Biolegend
CD19	BV711	SJ25C1	1:25	Biolegend

Supplementary Table 1. List of antibodies used in flow cytometry for the indicated analysis

**B cell subtypes**	**Marker Genes**	**Reference**
Activated naive	CD69, CCR7, CD83	(33,34)
Naïve	FCMR, HVCN1, SELL, IGHD	(34)
Germinal center	LM02, FGD6, MME, AICDA	(33)
Cycling	PCNA, TUBA1B, TOP2A	(33)
Memory	MS4A1, KLF2	(33,34)
Antibody secreting cells	XBP1, JCHAIN, MZB1, PRDM1	(34)

Supplementary Table 2. List of marker genes to identify B cell subpopulations

Antibody	Type	Clone	Provider	Fluorophore
CD3	Rabbit IgG	D7A6E	Cell Signaling Technology	OPAL520
PCK	Mix of mouse IgG1 and IgG2a	-	Sigma	OPAL480
CD19	Rabbit IgG	D4V4B	Cell Signaling Technology	OPAL650
CD11c	Rabbit IgG	D3V1E	Cell Signaling Technology	OPAL690
CD123	Rabbit IgG (polyclonal)	-	Thermo Fisher	OPAL540
CD38	Rabbit IgG	SP149	Roche	OPAL620
A2AR	Mouse IgG2a	7F6-G5-A2	Novus	OPAL570
MUM-1	Mouse IgG1	MUM1p	Dako	OPAL780

Supplementary Table 3. List of the antibodies and fluorophores used for the multiplex panel.
